# Antisocial personality disorder: what else can be done?

**DOI:** 10.1192/j.eurpsy.2022.1716

**Published:** 2022-09-01

**Authors:** V. Falcao, C. Pinto, M.J. Heitor

**Affiliations:** Hospital Beatriz Ângelo, Psychiatry And Mental Health Department, Loures, Portugal

**Keywords:** Treatment, Antisocial Personality disorder

## Abstract

**Introduction:**

Antisocial personality disorder (ASPD) is an under-researched mental disorder, and these patients are often excluded from mental healthcare and thus from studies. The consequences of antisocial behavior result in great burden for the patients, victims, family members and for society, and it is associated with criminality, substance use and relationship difficulties.

**Objectives:**

The aim of this abstract is to review the current possibilities of treatment, and its efficacy.

**Methods:**

We present a revision of the state of the art on treatment of ASPD, drawing from *PubMed* and using the keywords “Antisocial personality disorder” and “treatment”, in a time period ranging from 2011 to 2021, inclusive.

**Results:**

There is little evidence of effective treatments for patients with ASPD and no intervention has been established as the treatment of choice for this disorder. Recent studies, found benefits from Mentalization-based treatment (MBT), that specifically targets the ability to recognise and understand the mental states of oneself and others, an ability compromised in these patients. Specifically, reduction of anger, hostility, paranoia, and frequency of self-harm and suicide attempts, as well as the improvement of negative mood, general psychiatric symptoms, interpersonal problems, and social adjustment were found.

**Conclusions:**

ASPD is a condition that incurs substantial societal and individual costs. Although proper treatment is yet being discussed, MBT is a psychotherapeutic treatment that has shown some promising preliminary results. Thus, we believe that guidelines on the treatment of ASPD and possibilities for more systematical research, with prevention programs, is urgently needed.

**Disclosure:**

No significant relationships.

